# *Trans ε* viniferin decreases amyloid deposits and inflammation in a mouse transgenic Alzheimer model

**DOI:** 10.1371/journal.pone.0212663

**Published:** 2019-02-20

**Authors:** Martial Caillaud, Jérôme Guillard, Damien Richard, Serge Milin, Damien Chassaing, Marc Paccalin, Guylène Page, Agnès Rioux Bilan

**Affiliations:** 1 University of Poitiers, EA3808 Neurovascular Unit and Cognitive Disorders, Pôle Biologie Santé, POITIERS, France; 2 University of Poitiers, UMR CNRS 7285 Institute of Chemistry of Poitiers: Materials and Natural Resources, POITIERS, France; 3 Department of Pharmacology and biological Toxicology, UMR INSERM 1107, Clermont-Ferrand, France; 4 Poitiers University Hospital, Department of Pathology, Poitiers, France; 5 Poitiers University Hospital, Geriatrics Department, Poitiers, France; 6 Poitiers University Hospital, CMRR, Poitiers, France; Nathan S Kline Institute, UNITED STATES

## Abstract

As Alzheimer’s disease (AD) induces several cellular and molecular damages, it could be interesting to use multi-target molecules for therapeutics. We previously published that *trans ε*-viniferin induced the disaggregation of Aβ_42_ peptide and inhibited the inflammatory response in primary cellular model of AD. Here, effects of this stilbenoid were evaluated in transgenic APPswePS1dE9 mice. We report that *trans ε*-viniferin could go through the blood brain barrier, reduces size and density of amyloid deposits and decreases reactivity of astrocytes and microglia, after a weekly intraperitoneal injection at 10 mg/kg from 3 to 6 months of age.

## Introduction

Alzheimer’s disease (AD) affects many cellular and molecular targets and therefore requires multi-target molecules for therapeutics. Indeed, drugs currently proposed to treat AD do not prevent neurodegenerative processes and are only symptomatic therapies [1–3]. Thus, polyphenols presenting multiple pharmacological effects appear to be good potential candidates.

Polyphenols are natural substances obtained from plants, fruits and vegetables that can be found in beverages. Among them, resveratrol (3,5,4’-trihydroxy-trans-stilbene), a polyphenol of the stilbene family was studied for its effects in AD, because it was described as an anti-inflammatory drug [4], able to inhibit Aβ aggregation [5] and Aβ-induced neuronal apoptosis [6]. But this natural stilbenoid is very rapidly metabolized, and it would be necessary to administrate very high doses daily [7].

*Trans ε*-viniferin is a dehydrodimer of resveratrol. This natural stilbenoid, synthesized by *V*. *vinifera* in response to different kinds of stress can be constitutively found in the vine stalks and all woody parts of the vine. Its chemical structure corresponds to a dehydrodimer of resveratrol and could give superior properties than those observed with resveratrol, as well as being less metabolized.

Few findings concerning the role of *trans ε*-viniferin in AD have been published, except concerning its inhibitory role on Aβ aggregation [8–10]. But recently, we demonstrated that *trans ε-*viniferin both induced the disaggregation of Aβ_42_ peptide and inhibited inflammation on murine primary neuronal cultures with a higher efficiency than resveratrol [11].

The main objective of this work was to study the *trans ε*-viniferin action on size and density of amyloid deposits and neuroinflammation, using transgenic APPswePS1dE9 mice as *in vivo* model of AD.

## Material and methods

### Chemical products

Sodium fluoride (NaF), phenylmethylsulfonyl fluoride (PMSF), protease and phosphatase inhibitor cocktails, polyethylene glycol 200 (PEG 200), paraformaldehyde (PFA), 4’,6-diamnidino-2-phenylindole (DAPI) and all reagent-grade chemicals for buffers were purchased from Sigma (France). Sodium pentobarbital was obtained from CEVA, Animal Health (France), Quant-it protein assay and amyloid ELISA kit from Gibco-Invitrogen (Fisher Bioblock Scientific distributor, France) and guanidine from Acros Organics (USA). For immunofluorescence, monoclonal mouse antibody against amyloid peptide (clone W02), monoclonal rabbit antibody against GFAP and polyclonal goat anti-IBA-1were purchased from Millipore (France), Cell Signalling (Ozyme, France) and Abcam (France) respectively. Donkey anti-mouse-Alexa 488, donkey anti-rabbit-RRX, donkey anti-goat RRX and protease-Free Bovine Serum Albumin (BSA) were obtained from Jackson ImmunoResearch Europe Ltd (Interchim distributor, France). Absolute ethanol was purchased from Carlo Erba Reagents Rodano (France), Histosol plus from Shandon (France), and Stick On Q Path from VWR International.

### Animals and treatment

APPswePS1dE9 (B6C3F1, Stock # 004462) [12] and WT mice (B6C3F1, Stock # 10,010) were obtained from Jackson Laboratories (Bar Harbor, ME USA) and bred to create colonies of APPswePS1dE9 (Tg) and WT mice. This AD model develops amyloid deposits at 4–6 months, neuroinflammation and cognitive impairment at 12 months [13–15].

A total of 19 transgenic mice were included (9 males and 10 females). From 3 until 6 months of age, 9 mice (3 males and 6 females) received a weekly intraperitoneal (IP) injection of *trans ε*-viniferin at 10 mg/kg and 6 mice (3 males and 3 females) at 1 mg/kg, whereas 4 mice (3 males and 1 female) received the vehicle (polyethylene glycol 200: PEG 200) in 0.9% NaCl (PEG 200 at 0.44 mL/kg weight body). LD_50_ of PEG 200 for intraperitoneal injections in mouse is 7.5 mL/kg [16]. *Trans ε*-viniferin has been extracted and purified as previously described **[17]**. The use of animals was approved by the Ethical and Animal Care Committee (N°84 COMETHEA (Ethical Committee for Animal Experimentation) Poitou Charentes) and by the French ministry (agreement number: 376–201 5072717461 531). An agreement was obtained from The High Council of Biotechnology for transgenic animals in 2011 and renewed in 2015 (agreement number: 2040). All animal care and experimental procedures conformed with the French Decret number 2013–118, 1 February 2013 NOR: AGRG1231951D in accordance to European Community guidelines (directive 2010/63/UE). All efforts were made to minimize animal suffering, as well as the number of animals used. The animals were housed in a conventional state under adequate temperature (23 ± 3°C) and relative humidity (55 ± 5%) control with a 12/12 hour reversed light/dark with access to food and water *ad libitum*.

During all the study, the general state health of the mice was evaluated by monitoring their body weight, food and water intake. Plasma and organs were withdrawn in order to analyze biochemical parameters (AST, ALT, cholesterol, glycaemia, triglycerides) and macroscopic and microscopic studies respectively and no difference was found between both groups.

### Preparation of brain homogenates

A week after the last injection, at 6 months of age, mice were transcardially perfused with PBS (154 mM NaCl, 1.54 mM KH_2_PO_4_, 2.7 mM Na_2_HPO_4_.7H_2_O, pH 7.2) after deep anesthesia with 80 mg/kg IP pentobarbital. The brains were rapidly removed. The right hemisphere was immediately placed in 4% PFA overnight at 4°C for immunofluorescence studies. The left hemisphere was dissected and cortex and hippocampus were homogenized as previously described [18]. Briefly, cortex and hippocampus were homogenized using 10 up-and-down strokes of a prechilled Teflon-glass homogenizer in 20 volumes of lysis buffer (25 mM Tris-HCl, 150 mM NaCl, 1 mM EDTA, pH 7.4) and supplemented with 50 mM NaF, 1 mM PMSF, protease and phosphatase inhibitor cocktails (50 μL/g of tissue and 10 μL/mL of lysis buffer, respectively). Lysates were sonicated and centrifuged at 15,000*g* for 15 min at 4°C. The resulting supernatants were collected for Qubit protein assay according to the manufacturer’s protocol. Supernatants were stored at -80°C. For ELISA, pellet was suspended with 30 μL of supernatant before treatment of guanidine as explained below.

### Quantification of *trans ε*-viniferin

We have verified that this polyphenol goes through the blood brain barrier by detecting the level of *trans ε*-viniferin in brain after a weekly intraperitoneal injection. This detection was performed by CREPTA (Clermont-Ferrand, France).

For sample preparation: two stocks solution of viniferin were prepared at 1 g/L in methanol; one for standard calibration and the second for controls samples. All standards were stored at -20°C. Working solutions of viniferin were prepared at 5 μg/mL, 500 ng/mL, 50 ng/mL and 5 ng/mL in water. Four controls levels were used at 1, 8, 40 and 250 μg/L.

For cerebral cortex samples, eleven points of aqueous calibration curves were constructed in the concentration ranges of 0.1–250 μg/L in 500 μL of aqueous solution. Four controls were used to validate calibration curve. The analyses samples were prepared between 500 μL of homogenized cerebral cortex sample. Samples (standards, controls and biologics samples) were extracted with 2 mL of ethyl acetate / hexane (80/20, v/v). After sonication and vortex agitation, the organic phase was dissociated by centrifugation (3000 rpm/10min). Upper organic phases were collected, and evaporated under a stream of nitrogen at room temperature. The extracts were then reconstituted in 50μL of ammonium formiate buffer 0.5 mM / ACN (40/60, v/v) before HPLC analysis.

For liquid chromatography and mass spectrometry: 10 μL of standard or sample preparation were injected into liquid chromatography systems (Prominence UFLC Shimadzu, Marne la Vallée, France). Chromatographic separation was carried out using a reverse-phase column at 30°C using a Hypersil GOLD column (50 x 2.1 mm, 1.9 μm) (Thermo Fisher Scientific, San Jose, CA, USA). A gradient system with the mobile phase consisting of solvent A (0.1%, v/v; ammonium formiate 0.5 mM in water) and solvent B (0.1%, v/v; formic acid in acetonitrile) was used at a flow rate of 300 μL/min. Run time was set to 9.3 min. The auto sampler was kept at 4°C.

LC-MS/MS analyses were performed on a system of 5500 Qtrap quadripole linear trap mass spectrometer equipped with a turbo ionspray source operated in electrospray mode (ABSciex, Fester City, CA, USA). MS experiments were performed with a multiple reaction monitoring (MRM) condition in negative mode. For quantification of viniferin; one MRM transition was used (m/z 453.009 ➔ 385.1) and two other m/z confirmation: m/z for confirmation (453.009 ➔ 347.0 and 453.009 ➔ 359.0). Concentrations of viniferin in biological samples were determined by measuring of each chromatographic peak area using a calibration curve with a weight quadratic fit. Lower limit of quantification (LLOQ) for compound was 0.1 μg/L and upper limit of quantification (ULOQ) was 250 μg/L in sample.

Results showed that levels of viniferin were under the limit of quantification in PEG-treated mice while the mean was 2.27 ± 0.93 ng/mL in mice treated with 10 mg/kg/week during 3 months. Note that for 2 male mice, the level of viniferin was under LOQ.

### Aβ_42_ ELISA

Levels of Aβ_42_ were quantified using ELISA kit, as previously described [19] with minor modifications. Briefly, pellets obtained after preparation of hippocampal and cortical homogenates from left hemisphere as described above were suspended with 30 μL of supernatant containing soluble Aβ. Then, a homogenization with 8 volumes of guanidine–Tris buffer (5 M guanidine HCl/50 mM Tris–HCl, pH 8.0) was performed in order to extract insoluble Aβ. Homogenates were incubated at RT for 4 h before they were assayed. Samples were diluted in cold BSAT-DPBS reaction buffer (0.2 g/L KCl, 0.2 g/L KH_2_PO_4_, 8.0 g/L NaCl, 1.15 g/L Na_2_HPO_4_, 5% BSA, 0.03% Tween-20, pH 7.4) supplemented with Protease Inhibitor Cocktail. Samples were centrifuged at 16,000*g* for 20 minutes at 4°C. The supernatant was diluted in standard diluent buffer available in the kit. The final concentration of AEBSF (protease inhibitor in cocktail of proteases) was 1 mM in order to prevent proteolysis of Aβ peptides. The human Aβ42 standard was diluted in the same standard diluent buffer of samples. Plates were incubated with detection antibody overnight at 4°C. After washing, plates were incubated with HRP anti-rabbit antibody for 30 min at RT, then they were washed and stabilized chromogen was added in each well for 20 min in a dark chamber at RT. After stopping the reaction, the absorbance of plates was read at 450 nm using the Multiskan spectrum spectrophotometer. The standard curves were established using a range of concentrations (15.63–1,000 pg/mL) of a synthetic Aβ_42_ peptide. Data are expressed as pg of total Aβ_42_/mg of proteins.

### Immunofluorescence

After 24 hours in 4% PFA at 4°C, right brain hemispheres were rinsed in PBS, dehydrated, and embedded in paraffin for sagittal sectioning (4 μm in thickness). Sagittal sections were cut in a microtome (Microm HM335E) and thaw mounted on Super-Frost Plus1 slides (CML, Nemours, France) with albumin solution (Stick On Q Path) and conserved at 4°C until their utilization. Immunolabellings were performed as previously described [19, 20]. Multiple labelled samples (3 slices *per* mice) were examined with Olympus BX51 epifluorescent optical microscope. Images were blind analyzed with ImageJ. For cortex, frontal and parietotemporal areas have been analyzed and for hippocampus, dentate gyrus and CA1 area. For the analysis with ImageJ, the brightness was adjusted at 50 for all the photos. For quantification of global amyloid, GFAP or IBA-1 signals, all densities indicated by « raw integrated density » were compiled in Graph Pad Prism for statistical analysis. However, to quantify signals of “only amyloid deposits”, particles with size superior to 100 pixels^2^ were only analyzed. Then all particles corresponding to intracellular labelling were excluded. Finally, all Raw Integrated Densities of conserved particles were added and compiled in Graph Pad Prism for statistical analysis.

### Statistical analysis

Results are expressed as means ± standard error (SEM). To compare quantitative variables between two groups of mice, Mann-Whitney tests were used, using the statistical program GraphPad Instat (GraphPad Software, San Diego, CA, USA). The level of significance was p < 0.05.

## Results and discussion

### Decrease in amyloid deposits by *trans ε*-viniferin without reduction of amyloid load

**Fig 1:**
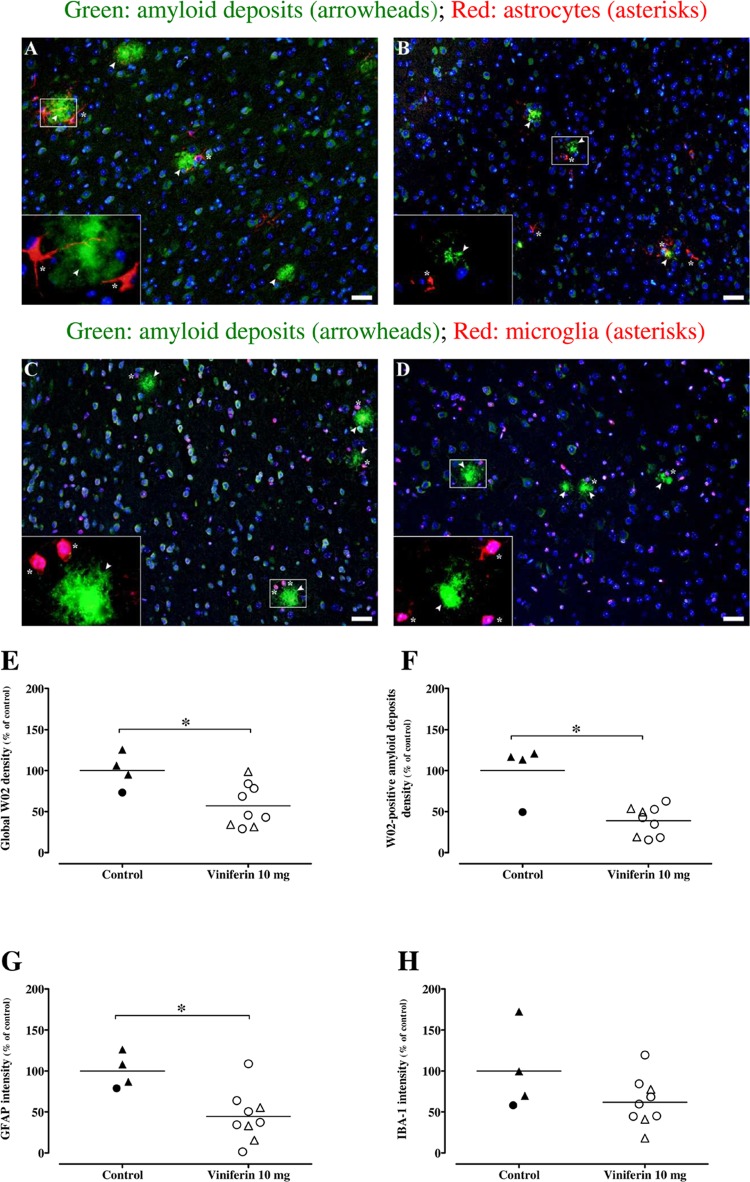
Effects of *trans ε*-viniferin at 10 mg/kg on amyloid deposits and neuroinflammation in cortex. The double transgenic APPswePS1dE9 mice were treated by *trans ε*-viniferin (10 mg/kg) or its vehicle (PEG 200) intraperitoneally from 3 to 6 months of age. Then, immunofluorescence staining was performed as described in method section. Paraffined and sagittal sections (4 μm in thickness) were incubated with monoclonal mouse antibody against amyloid peptide (clone W02) and monoclonal rabbit antibody against GFAP for astrocyte detection (representative images of frontal cortex in panels A for vehicle and B for *trans ε*-viniferin, respectively) or polyclonal goat anti-IBA-1 for microglia detection (representative images of frontal cortex in panels C for vehicle and D for *trans ε*-viniferin, respectively). Donkey anti-mouse-Alexa 488 (green channel) and donkey anti-rabbit-RRX or donkey anti-goat RRX (red channel) were used as secondary antibodies, respectively. Nuclei were stained with DAPI (blue channel). Scale bars: 50 μm. On each image, a magnification (X10) of the amyloid plaque delineated by a white frame was added. Signal of W02 throughout frontal and parietotemporal cortex and only in amyloid deposits, GFAP and IBA-1 signals were quantified by using image J software 1.47 V and raw integrated densities were represented in panels E, F, G and H, respectively. The line represents the mean of 4 to 9 mice in each group, expressed as percentage of control (rounds represent females, triangles represent males). To compare values between untreated APPswePS1dE9 mice and APPswePS1dE9 mice treated with *trans ε*-viniferin, Mann-Whitney test was used. *p < 0.05 compared to respective controls.

**Fig 2:**
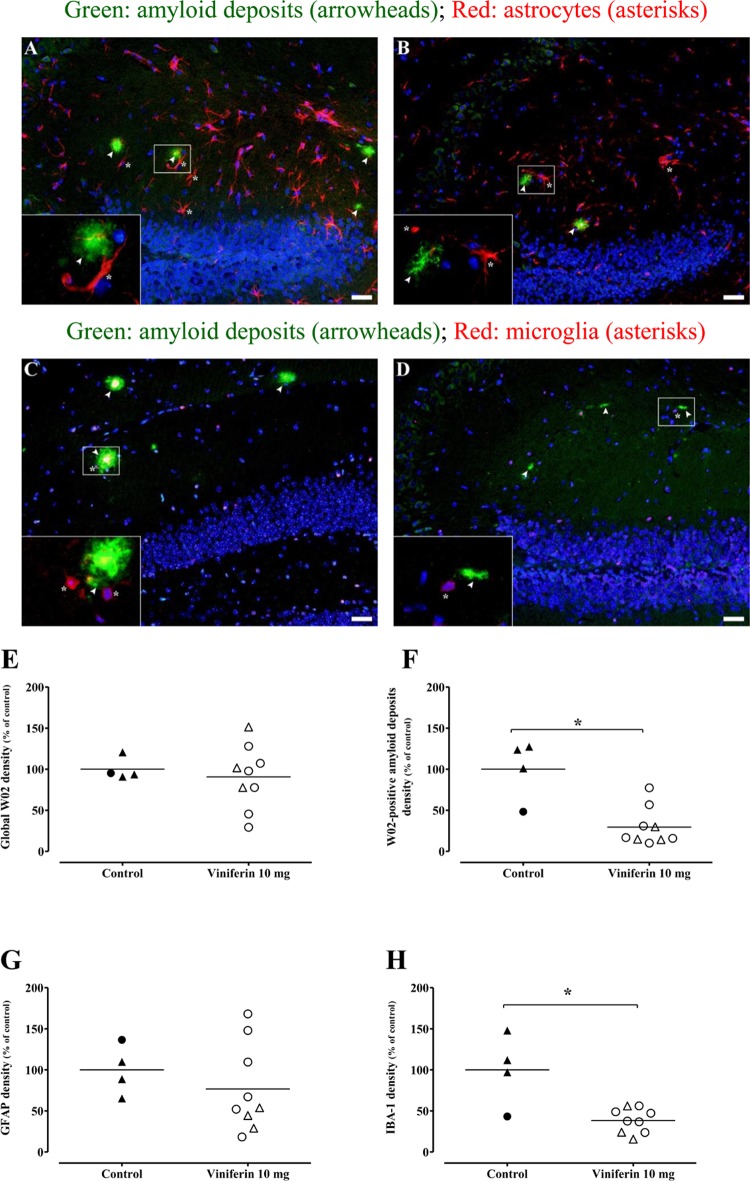
Effects of *trans ε*-viniferin at 10 mg/kg on amyloid deposits and neuroinflammation in hippocampus. The double transgenic APPswePS1dE9 mice were treated by *trans ε*-viniferin (10 mg/kg) or its vehicle (PEG 200) intraperitoneally from 3 to 6 months of age. Then, immunofluorescence staining was performed as described in method section. Paraffined and sagittal sections (4 μm in thickness) were incubated with monoclonal mouse antibody against amyloid peptide (clone W02) and monoclonal rabbit antibody against GFAP for astrocyte detection (representative images of dentate gyrus in panels A for vehicle and B for *trans ε*-viniferin, respectively) or polyclonal goat anti-IBA-1 for microglia detection (representative images of dentate gyrus in panels C for vehicle and D for *trans ε*-viniferin, respectively). Donkey anti-mouse-Alexa 488 (green channel) and donkey anti-rabbit-RRX or donkey anti-goat RRX (red channel) were used as secondary antibodies, respectively. Nuclei were stained with DAPI (blue channel). Scale bars: 50 μm. On each image, a magnification (X10) of the amyloid plaque delineated by a white frame was added. Quantifications of global signal of W02 throughout CA1 and dentate gyrus hippocampal regions and only in amyloid deposits, GFAP and IBA-1 signals were performed by using image J software 1.47 V and raw integrated densities were represented respectively in panels E, F, G and H. The line represents the mean of 4 to 9 mice in each group, expressed as percentage of control (rounds represent females, triangles represent males). To compare values between untreated APPswePS1dE9 mice and APPswePS1dE9 mice treated with *trans ε*-viniferin, Mann-Whitney test was used. *p < 0.05, compared to respective controls.

This AD model develops amyloid deposits detectable from the age of 4 months [13, 14]. Amyloid deposits in both cortex and hippocampus of each mouse were visualized, using monoclonal mouse anti-amyloid W02 clone, which recognizes amino acid residues 4–10 of human Aβ (Figs 1 and 2). *Trans ε*-viniferin at 10 μM induced a reduction of size and density amyloid deposits (showed by arrowheads) compared to control in cortex (Fig 1B and 1D *versus* Fig 1A and 1C, respectively). Quantification of this amyloid signal in the whole area showed a significant decrease in the cortex of viniferin-treated mice (decrease by 43%, p<0.05, as shown in Fig 1E) and no difference in the hippocampus (Fig 2E), compared to vehicle-treated mice.

Immunofluorescence makes it possible to detect any W02-positive pixel, whatever its soluble or insoluble form of amyloid peptide, however the sensitivity is less than that obtained with an absolute quantification according to a standard curve. Note that this antibody which recognizes amino acid residues 4–10 of human Aβ, binds also to the precursor to amyloid peptide (APP) or its oligomers.

**Fig 3:**
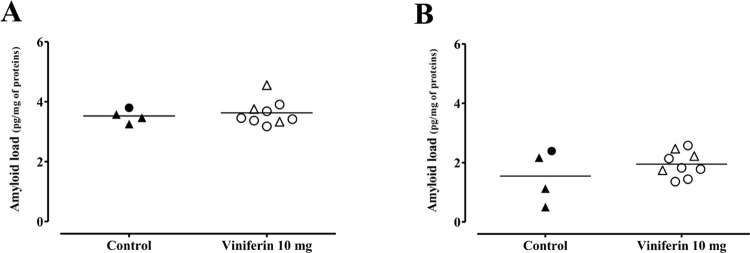
Effects of *trans ε*-viniferin at 10 mg/kg on amyloid load. For each group of mice, levels of Aβ_42_ in cortex (Fig 3A) and in hippocampus (Fig 3B) were quantified using ELISA kit. The line represents the mean of 4 to 9 mice in each group, expressed as pg of Aβ_42_/mg of proteins. To compare values between untreated APPswePS1dE9 mice and APPswePS1dE9 mice treated with *trans ε*-viniferin, Mann-Whitney test was used but no statistical difference was observed.

We therefore wished to complete this result by quantifying the total amyloid load by an ELISA test. The results showed that in the cortex (3.63 ± 0.14 pg/mg of proteins in viniferin-treated mice *versus* 3.52 ± 0.11 pg/mg of proteins in control mice) and in the hippocampus, (1.95 ± 0.14 pg/mg of proteins in viniferin-treated mice *versus* 1.55 ± 0.45 pg/mg of proteins in control mice) the total amyloid load is identical between the treated and control mice (Fig 3A and 3B, respectively). ELISA quantifies the absolute total Aβ_42_ rate.

When image analysis was focused on aggregated amyloid plaques, results showed that *trans ε*-viniferin has a significant inhibitory effect on the amyloid fibrillary deposits. Indeed, results showed a significant decrease by 61% in the cortex (p<0.05; Fig 1F) and by 70% in the hippocampus (p<0.05; Fig 2F), compared to controls. These results suggest that *trans ε*-viniferin induces an inhibition of amyloid aggregation, both in male and female mice, but without rescuing amyloidogenesis. Previous studies showed physical interactions between amyloid peptide and *trans ε*-viniferin [8], suggesting a mechanism of the inhibition of Aβ aggregation physically linked to *trans ε*-viniferin.

Differences between immunofluorence using clone W02 and Aβ_42_ ELISA may have at least three explanations: **(i)** in these transgenic mice, the clone W02 antibody greatly stains amyloid plaques with a weak intracellular staining, suggesting that the soluble Aβ species or their soluble oligomers were under estimated by immunofluorescence compared to ELISA, **(ii)** furthermore, ELISA kit specifically quantifies Aß_42_ while W02 antibody detects all amino acid residues 4–10 of human Aβ species. It is known that these transgenic mice largely produce Aß_40_ [21, 22], **(iii)** other authors also observed these differences between immunostaining of amyloid burden and total levels of Aβ_42_ and Aβ_40_ by ELISA in a program of immunization of transgenic mice TgCRND8. They indicated that their antibodies induced in these mice may bind to β-sheet oligomeric aggregates and inhibit further assembly. Thus, this redistribution of Aβ from dense-cored plaques to diffuse Aβ deposits needs not necessarily cause large changes in total cerebral Aβ [23]. One may propose that *trans ε*-viniferin could also inhibit assembly of Aβ oligomeric protofibrils.

It should be noted that *trans ε*-viniferin at only 1 mg/kg did not have effect (S1 and S2 Figs).

### Anti-inflammatory effects of *trans ε*-viniferin

In this AD model, a robust neuroinflammatory response is present at 12 months [18, 19]. However, around 6 months of age, a slight inflammation is detectable, resulting in an activation of cellular actors (astrocytes and microglia) and an expression of pro-inflammatory factors [13, 24, 25].

This inflammatory response is a double-edged sword [26, 27]. At the beginning, it is a self-defense reaction, starting as soon as there is a threshold of accumulated amyloid peptides. This early inflammatory response would represent a protective reaction to neurodegeneration. Then, the late process of neuroinflammation, initiating as soon as the first amyloid plaques are established would correspond to a destructive process that would contribute to further loss of brain function.

In transgenic APPswePS1dE9 mice, limited early deposits around 4 months of age were detected although by 6 months plaques were easily detectable [13, 14]. In our study, the treatment with *trans ε*-viniferin started at 3 months before amyloid deposits.

*Trans ε*-viniferin has induced changes in appareance of astrocytes (showed by asterisks Fig 1B *versus* Fig 1A). Indeed, in the cortex of vehicle-treated mice, astrocytes around the amyloid plaques, are hypertrophic and show a exacerbed star structure. In the cortex of mice treated with *trans ε*-viniferin, astrocytes are not hypertophic. However clustered amoeboid microglia (showed by asterisks Fig 1D *versus* Fig 1C) were associated with amyloid plaques in cortex in both groups of mice. On the contrary, in hippocampus, few microglia were observed around amyloid plaques (Fig 2D *versus* Fig 2C).

The reactivity of astrocytes and microglia has been evaluated by quantify the labellings of GFAP and IBA-1, respectively. Indeed, these biomarkers are described as over-expressed in these cells in an environment with stress (here amyloid peptide) and inflammation. Quantification of GFAP signal showed a significant decrease in cortex (decrease by 55%, p<0.05 as shown in Fig 1G) and no difference in hippocampus (decrease by 23%, Fig 2G). Quantification of IBA-1 signal showed a decrease both in the cortex (not significant decrease by 43%, as shown in Fig 1H) and in the hippocampus (significant decrease by 62%, p<0.05, Fig 2H). These results suggest that *trans ε*-viniferin partially decreased the reactivity of astrocytes and microglia at 6 months of age.

It is known that amyloid plaques and its associated inflammatory response develop at early stage of the life of APPswe/PS1dE9 mice and progressively increase with age [13, 24]. The role of *trans ε*-viniferin by inhibiting Aβ aggregation [8–10] and by inducing the disaggregation of Aβ_42_ peptide [11] may explain the consequent decrease in inflammatory response. But its direct effect on inflammatory signalling pathways remains to be demonstrated.

All these results suggest that *trans ε*-viniferin decreases the formation of amyloid plaques in this AD model at nontoxic concentration (10 mg/kg). In a similar manner, the first activation of cellular inflammatory actors is slighty reduced. These promising results remain to be now confirmed over a longer period (up to 12 months). Moreover, beneficial effects of *trans ε*-viniferin on cognitive impairment could be evaluated using behavioural analysis such as Morris water maze.

## Supporting information

S1 Fig Effects of *trans ε*-viniferin at 1 mg/kg on amyloid deposits and neuroinflammation in cortex. The double transgenic APPswePS1dE9 mice were treated by *trans ε*-viniferin (1 mg/kg) or its vehicle (PEG 200) intraperitoneally from 3 to 6 months of age. Then, immunofluorescence staining was performed as described in the method section. Paraffined and sagittal sections (4 μm in thickness) were incubated with monoclonal mouse antibody against amyloid peptide (clone W02) and monoclonal rabbit antibody against GFAP for astrocyte detection (representative images of frontal cortex in panels A for vehicle and B for *trans ε*-viniferin, respectively) or polyclonal goat anti-IBA-1 for microglia detection (representative images of frontal cortex in panels C for vehicle and D for *trans ε*-viniferin, respectively). Donkey anti-mouse-Alexa 488 (green channel) and donkey anti-rabbit-RRX or donkey anti-goat RRX (red channel) were used as secondary antibodies, respectively. Nuclei were stained with DAPI (blue channel). Scale bars: 50 μm. On each image, a magnification (X10) of the amyloid plaque delineated by a white frame has been added. Signal of W02 throughout frontal and parietotemporal cortex and only in amyloid deposits, GFAP and IBA-1 signals have been quantified by using image J software 1.47 V and raw integrated densities were represented in panels E, F, G and H, respectively. The line represents the mean of 4 to 6 mice in each group, expressed as percentage of control (rounds represent females, triangles represent males). To compare values between untreated APPswePS1dE9 mice and APPswePS1dE9 mice treated with *trans ε*-viniferin, Mann-Whitney test was used but no statistical difference was observed.
(TIF)

S2 Fig Effects of *trans ε*-viniferin at 1 mg/kg on amyloid deposits and neuroinflammation in hippocampus. The double transgenic APPswePS1dE9 mice were treated by *trans ε*-viniferin (1 mg/kg) or its vehicle (PEG 200) intraperitoneally from 3 to 6 months of age. Then, immunofluorescence staining was performed as described in the method section. Paraffined and sagittal sections (4 μm in thickness) were incubated with monoclonal mouse antibody against amyloid peptide (clone W02) and monoclonal rabbit antibody against GFAP for astrocyte detection (representative images of dendate gyrus in panels A for vehicle and B for *trans ε*-viniferin, respectively) or polyclonal goat anti-IBA-1 for microglia detection (representative images of dendate gyrus in panels C for vehicle and D for *trans ε*-viniferin, respectively). Donkey anti-mouse-Alexa 488 (green channel) and donkey anti-rabbit-RRX or donkey anti-goat RRX (red channel) were used as secondary antibodies, respectively. Nuclei were stained with DAPI (blue channel). Scale bars: 50 μm. On each image, a magnification (X10) of the amyloid plaque delineated by a white frame has been added. Quantifications of global signal of W02 throughout CA1 and dentate gyrus hippocampal regions and only in amyloid deposits, GFAP and IBA-1 signals were performed by using image J software 1.47 V and raw integrated densities were represented respectively in panels E, F, G and H. The line represents the mean of 4 to 6 mice in each group, expressed as percentage of control (rounds represent females, triangles represent males). To compare values between untreated APPswePS1dE9 mice and APPswePS1dE9 mice treated with *trans ε*-viniferin, Mann-Whitney test was used but no statistical difference was observed.
(TIF)

S3 Fig Effects of *trans ε*-viniferin at 1 mg/kg on amyloid load. For each group of mice, levels of Aβ_42_ in the cortex (S3A Fig) and in the hippocampus (S3B Fig) were quantified using ELISA kit. The line represents the mean of 4 to 6 mice in each group, expressed as pg of Aβ_42_/mg of proteins. To compare values between untreated APPswePS1dE9 mice and APPswePS1dE9 mice treated with *trans ε*-viniferin, Mann-Whitney test was used but no statistical difference was observed.
(TIF)

## Abbreviations

AD: Alzheimer’s disease
Aβ: amyloid β
PEG 200: polyethylene glycol 200
