# Supplementary figures and images for: Trans ε viniferin decreases amyloid deposits and inflammation in a mouse transgenic Alzheimer model
Source: PLoS One. 2019 Feb 20;14(2):e0212663. doi: 10.1371/journal.pone.0212663 (PMC6382128; doi:10.1371/journal.pone.0212663)

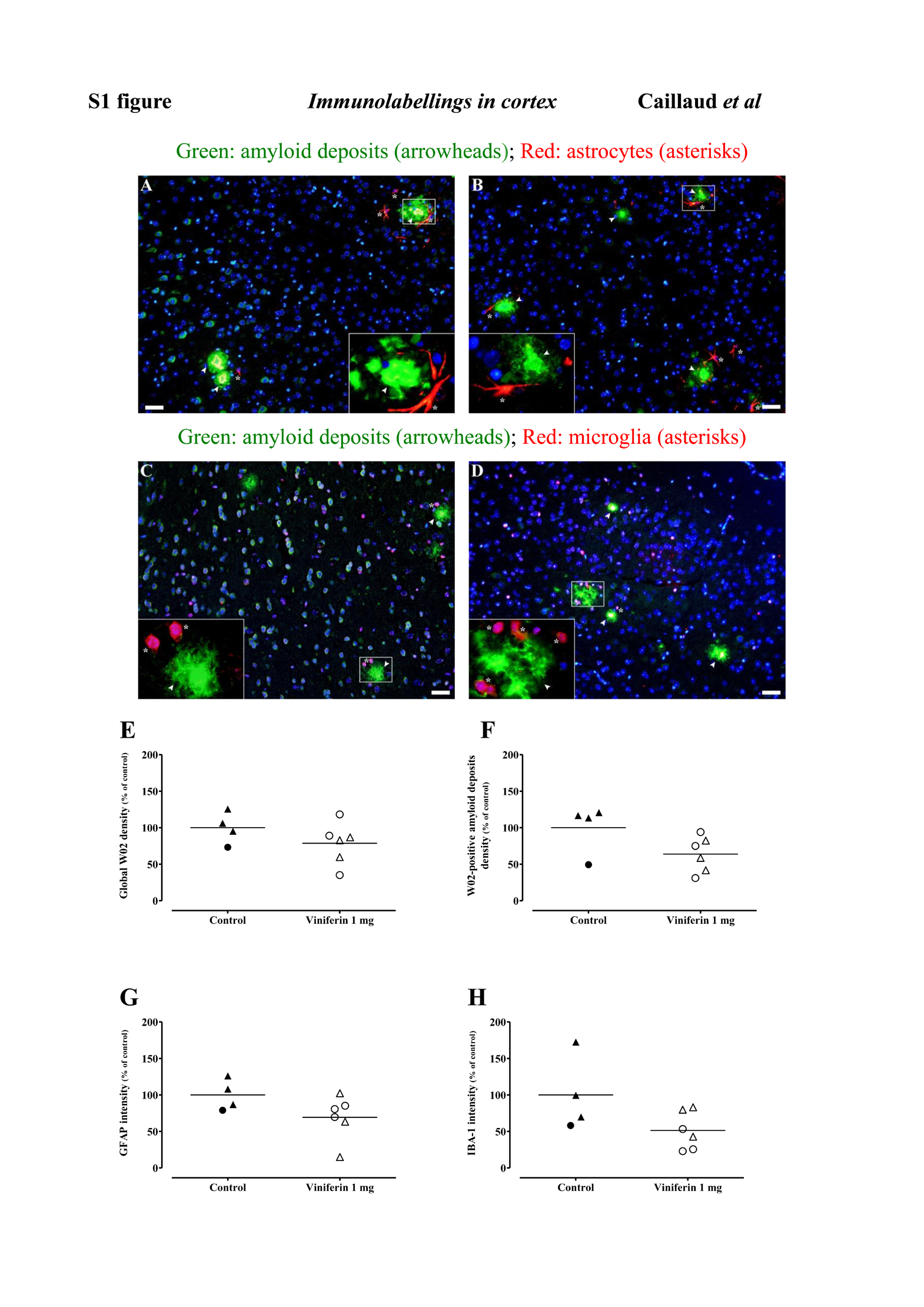

Supplement: S1 Fig — The double transgenic APPswePS1dE9 mice were treated by trans ε-viniferin (1 mg/kg) or its vehicle (PEG 200) intraperitoneally from 3 to 6 months of age. Then, immunofluorescence staining was performed as described in the method section. Paraffined and sagittal sections (4 μm in thickness) were incubated with monoclonal mouse antibody against amyloid peptide (clone W02) and monoclonal rabbit antibody against GFAP for astrocyte detection (representative images of frontal cortex in panels A for vehicle and B for trans ε-viniferin, respectively) or polyclonal goat anti-IBA-1 for microglia detection (representative images of frontal cortex in panels C for vehicle and D for trans ε-viniferin, respectively). Donkey anti-mouse-Alexa 488 (green channel) and donkey anti-rabbit-RRX or donkey anti-goat RRX (red channel) were used as secondary antibodies, respectively. Nuclei were stained with DAPI (blue channel). Scale bars: 50 μm. On each image, a magnification (X10) of the amyloid plaque delineated by a white frame has been added. Signal of W02 throughout frontal and parietotemporal cortex and only in amyloid deposits, GFAP and IBA-1 signals have been quantified by using image J software 1.47 V and raw integrated densities were represented in panels E, F, G and H, respectively. The line represents the mean of 4 to 6 mice in each group, expressed as percentage of control (rounds represent females, triangles represent males). To compare values between untreated APPswePS1dE9 mice and APPswePS1dE9 mice treated with trans ε-viniferin, Mann-Whitney test was used but no statistical difference was observed. (TIF) [file pone.0212663.s001.tif]

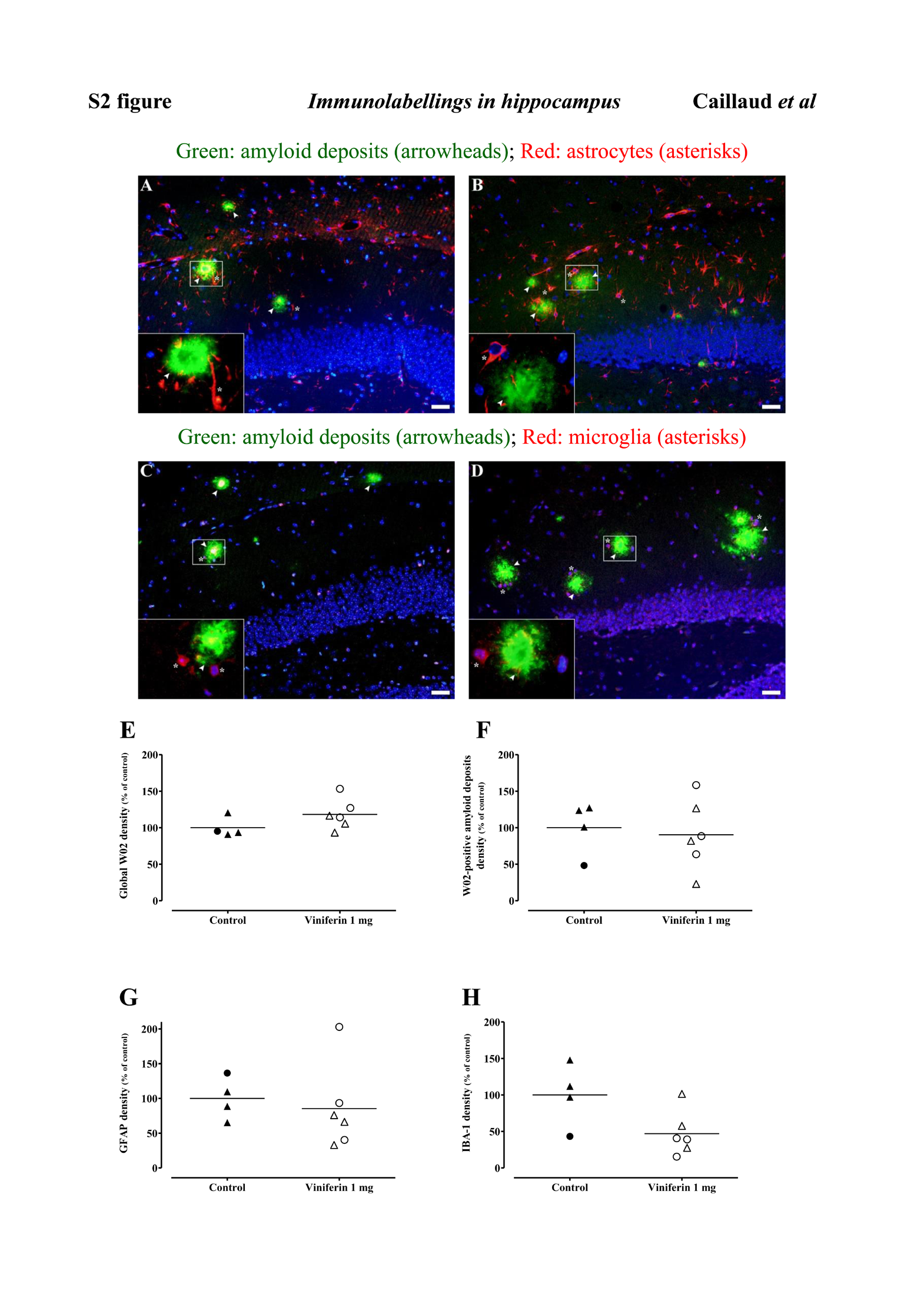

Supplement: S2 Fig — The double transgenic APPswePS1dE9 mice were treated by trans ε-viniferin (1 mg/kg) or its vehicle (PEG 200) intraperitoneally from 3 to 6 months of age. Then, immunofluorescence staining was performed as described in the method section. Paraffined and sagittal sections (4 μm in thickness) were incubated with monoclonal mouse antibody against amyloid peptide (clone W02) and monoclonal rabbit antibody against GFAP for astrocyte detection (representative images of dendate gyrus in panels A for vehicle and B for trans ε-viniferin, respectively) or polyclonal goat anti-IBA-1 for microglia detection (representative images of dendate gyrus in panels C for vehicle and D for trans ε-viniferin, respectively). Donkey anti-mouse-Alexa 488 (green channel) and donkey anti-rabbit-RRX or donkey anti-goat RRX (red channel) were used as secondary antibodies, respectively. Nuclei were stained with DAPI (blue channel). Scale bars: 50 μm. On each image, a magnification (X10) of the amyloid plaque delineated by a white frame has been added. Quantifications of global signal of W02 throughout CA1 and dentate gyrus hippocampal regions and only in amyloid deposits, GFAP and IBA-1 signals were performed by using image J software 1.47 V and raw integrated densities were represented respectively in panels E, F, G and H. The line represents the mean of 4 to 6 mice in each group, expressed as percentage of control (rounds represent females, triangles represent males). To compare values between untreated APPswePS1dE9 mice and APPswePS1dE9 mice treated with trans ε-viniferin, Mann-Whitney test was used but no statistical difference was observed. (TIF) [file pone.0212663.s002.tif]

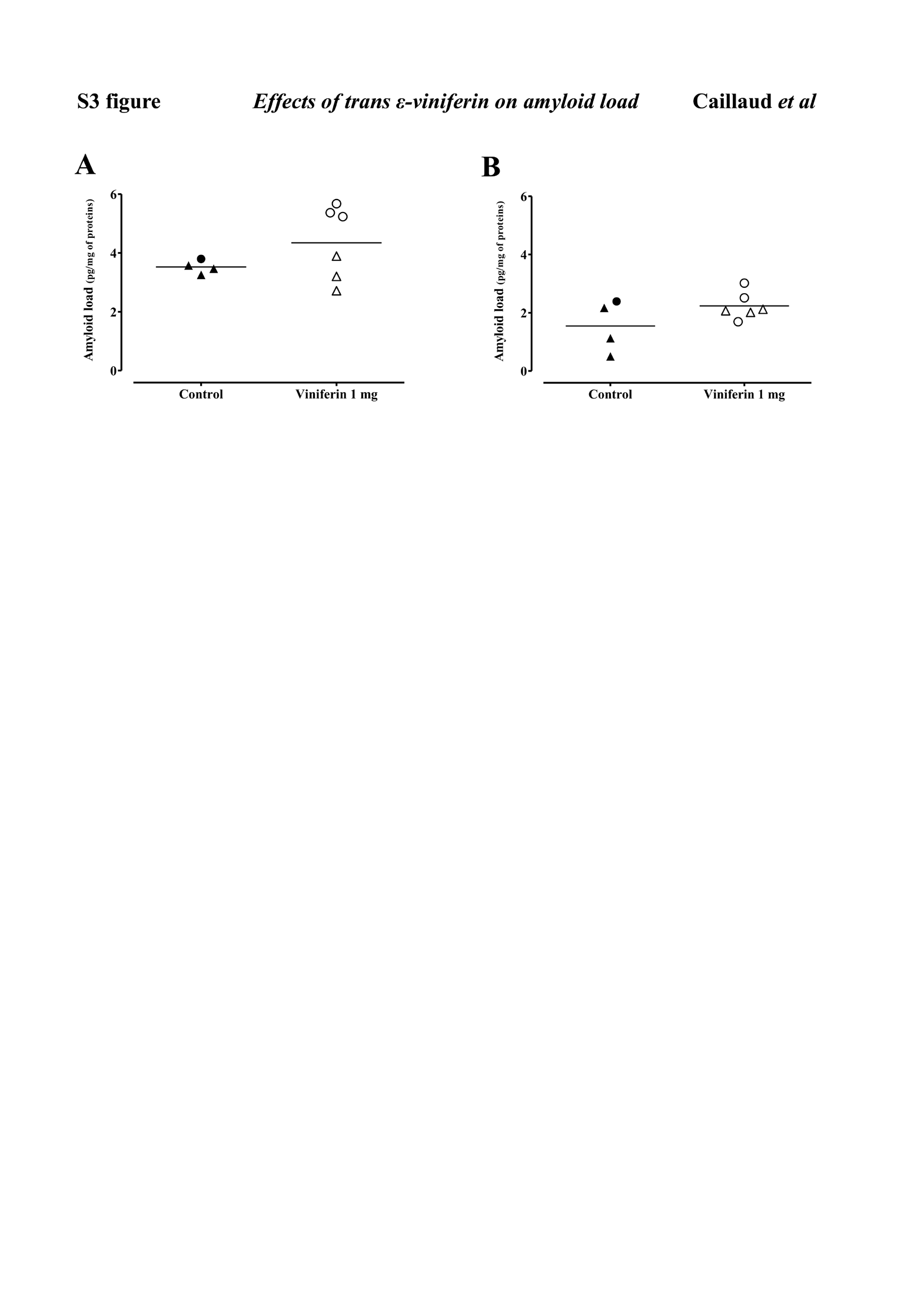

Supplement: S3 Fig — For each group of mice, levels of Aβ42 in the cortex (S3A Fig) and in the hippocampus (S3B Fig) were quantified using ELISA kit. The line represents the mean of 4 to 6 mice in each group, expressed as pg of Aβ42/mg of proteins. To compare values between untreated APPswePS1dE9 mice and APPswePS1dE9 mice treated with trans ε-viniferin, Mann-Whitney test was used but no statistical difference was observed. (TIF) [file pone.0212663.s003.tif]
